# A Common Variant Of Ubiquinol-Cytochrome c Reductase Complex Is Associated with DDH

**DOI:** 10.1371/journal.pone.0120212

**Published:** 2015-04-07

**Authors:** Ye Sun, Cheng Wang, Zheng Hao, Jin Dai, Dongyang Chen, Zhihong Xu, Dongquan Shi, Ping Mao, Huajian Teng, Xiang Gao, Zhibin Hu, Hongbing Shen, Qing Jiang

**Affiliations:** 1 The Center of Diagnosis and Treatment for Joint Disease, Drum Tower Hospital Affiliated to Medical School of Nanjing University, Nanjing, Jiangsu 210008, China; 2 Laboratory for Bone and Joint Diseases, Model Animal Research Center, Nanjing University, Nanjing, Jiangsu 210061, China; 3 MOE Key Laboratory of Model Animal for Disease Study, Model Animal Research Center, Nanjing University, Nanjing, Jiangsu 210061, China; 4 Department of Epidemiology and Biostatistics, School of Public Health, Nanjing Medical University, Nanjing, China; Central China Normal University, CHINA

## Abstract

**Purpose:**

Genetic basis of Developmental dysplasia of the hip (DDH) remains largely unknown. To find new susceptibility genes for DDH, we carried out a genome-wide association study (GWAS) for DDH.

**Methods:**

We enrolled 386 radiology confirmed DDH patients and 558 healthy controls (Set A) to conduct a genome-wide association study (GWAS). Quality-control was conducted at both the sample and single nucleotide polymorphism (SNP) levels. We then conducted a subsequent case-control study to replicate the association between a promising loci, rs6060373 in *UQCC* gene and DDH in an independent set of 755 cases and 944 controls (set B).

**Results:**

In the DDH GWAS discovering stage, 51 SNPs showed significance of less than 10^-4^, and another 577 SNPs showed significance of less than 10-3. In *UQCC*, all the 12 genotyped SNPs showed as promising risk loci. Genotyping of rs6060373 in set A showed the minor allele A as a promising risk allele (p = 4.82*10^-7^). In set A, the odds ratio of allele A was 1.77. Genotyping of rs6060373 in Set B produced another significant result (p = 0.0338) with an odds ratio of 1.18 for risk allele A. Combining set A and set B, we identified a total p value of 3.63*10^-6^ with the odds ratio of 1.35 (1.19–1.53) for allele A.

**Conclusion:**

Our study demonstrates common variants of *UQCC*, specifically rs6060373, are associated with DDH in Han Chinese population.

## Introduction

Developmental dysplasia of the hip (DDH, OMIM #142700) is one common skeletal disorder, presenting with shallow acetabulum and decreased coverage of the femoral head. [1] Incidence of DDH varies from 0.1% to 1.84% in Caucasian population, and 0.1%-0.5% in Chinese population. [2] Persistent DDH can induce chronic hip pain, dysfunction and increase the hip osteoarthritis risk. [3]

DDH is a polygenic disease with both environmental and genetic risk factors. [4] Though Mechanical factors (e.g. breech delivery, high birth weight, primiparity and oligoamnios) are suggested [5,6], it is accepted that genetic components are a crucial part in the etiology of DDH. Several DDH susceptibility genes (e.g. *GDF5*, *TBX4*, *ASPN* and *PAPPA2*) were discovered by association study in Chinese and Caucasian populations [7–10]. However, the genetic basis of DDH remains largely unknown.

Genome-wide association study (GWAS) is a genetic method for explaining complex human diseases such as osteoarthritis [11,12]. GWAS has the potential to identify new susceptibility genes with previously unknown function and their relationship to the disorder. Susceptibility genes for several common skeletal disorders have been identified by using this approach[11–13]. In order to find new susceptibility genes for DDH, we carried out a genome-wide association study for DDH. Within our GWAS result, we found 12 variants in Ubiquinol-cytochrome c reductase complex chaperone (*UQCC*) gene associated with DDH (Table 1).

**Table 1:** Identified SNPs in *UQCC* gene in the genome-wide analysis.

		Case Genotype					Control Genotype					Test for allele frequency	
dbSNP ID	Chrome Position	11	12	22	Sum	Allele 2 frequency	11	12	22	Sum	Allele 2 frequency	P Value	Odds ratio(95% CI)
**rs6060355**	33890061	259	110	17	386	0.187	286	226	46	558	0.285	0.00000104	1.74(1.39–2.17)
**rs878639**	33894463	259	110	17	386	0.187	284	228	46	558	0.287	0.00000349	1.75(1.40–2.91)
**rs1406948**	33905619	260	109	17	386	0.185	286	226	46	558	0.285	0.00000396	1.75(1.40–2.19)
**rs6088791**	33907909	260	109	17	386	0.185	285	226	46	557	0.285	0.000000657	1.76(1.41–2.20)
**rs6060371**	33913322	260	107	18	385	0.186	286	225	47	558	0.286	0.000000693	1.75(1.40–2.19)
**rs6060373**	33914208	260	109	17	386	0.185	285	226	47	558	0.287	0.000000482	1.77(1.41–2.21)
**rs4911178**	33952620	258	111	17	386	0.188	285	227	46	558	0.286	0.00000119	1.73(1.39–2.16)
**rs60696658**	33954913	258	110	17	385	0.187	286	223	48	557	0.286	0.000000877	1.74(1.40–2.18)
**rs4911494**	33971914	259	110	17	386	0.187	286	226	46	558	0.285	0.00000104	1.74(1.39–2.17)
**rs6088813**	33975181	259	110	17	386	0.187	284	226	46	556	0.286	0.000000832	1.75(1.40–2.18)
**rs6087704**	34001058	259	110	17	386	0.187	286	226	46	558	0.285	0.00000104	1.74(1.39–2.17)
**rs6087705**	34001250	259	110	17	386	0.187	286	226	46	558	0.285	0.00000104	1.74(1.39–2.17)

Population set A was genotyped. Allele 1 and allele 2 indicate the major and minor allele in the DDH population, respectively, and 11, 12 and 22 indicate homozygote of allele 1 and heterozygote and homozygote of allele 2, respectively. Odds ratio shown is for allele 1 versus allele 2.

*UQCC* encodes a zinc-binding protein, putatively repressed by fibroblast growth factor 2 (*FGF2*), which functions with several genes in morphogenesis and growth of skeleton. [14,15] *UQCC* is expressed in differentiating chondrocytes,[16] and is first expressed at early stages of mesenchymal cell proliferation in mouse.[17] *UQCC* has been reported as an important candidate gene in genome-wide association studies for spine bone size, height and testicular germ cell tumors.[18–20]

Based on the importance of *UQCC* in chondrogenesis, we thought *UQCC* could be an attractive candidate gene of DDH, and then conducted a subsequent case-control study to evaluate the association between *UQCC* gene and DDH, and found *UQCC* was associated with DDH

## Materials and Methods

### Patients

We enrolled 386 radiology confirmed DDH patients and 558 healthy controls (Set A) to conduct a case-control genome-wide association study (GWAS). DDH patients were consecutively recruited from the Center of Diagnosis and Treatment for Development dysplasia of hip, Kang’ai Hospital. Controls were recruited from the First Affiliated Hospital of Nanjing Medical University and the Affiliated Nanjing Children's Hospital of Nanjing Medical University (Nanjing, China) between March 2006 and March 2009.

An independent set of up to 755 cases and 944 controls (set B) were applied for replication of the most promising loci. DDH patients were also consecutively recruited from the Center of Diagnosis and Treatment for Development dysplasia of hip, Kang’ai Hospital. Controls were enrolled at the Physical Examination Center, Drum Tower Hospital, affiliated to the Medical School of Nanjing University. The diagnosis of DDH was made on the basis of clinical criteria and radiographic evidence by experts. All controls had no symptom or history of DDH. Subjects with any systemic syndrome were excluded. All the subjects were Han Chinese living in or around Nanjing. The study was approved by the ethical committee of the Nanjing University and the ethical committee of Nanjing Medical University, and written informed consent was obtained from all patients and controls. Written informed consent was obtained from guardians on behalf of the minors/children enrolled in this study.

### Methods

DNA was extracted from all the subjects either from peripheral blood using the NucleoSpin Blood QuickPure Kit (Macherey-Nagel GmbH & Co. KG, Düren, German) or buccal swabs using the DNA IQ System (Promega, Madison, WI) according to the manufacture’s protocol. The set A samples were genotyped by using Illumina Human Omni ZhongHua-8BeadChips (Illumina, San Diego, CA, USA). Quality-control was conducted at both the sample and single nucleotide polymorphism (SNP) levels (S1 Fig). We performed case-control analysis for all the SNPs in set A. From the primary result, we found a region at 20q11.22 was significantly associated with DDH. 21 SNPs in the region showed significance. To check the most promising loci, set B samples were genotyped by Taqman assay. The samples were genotyped by laboratory personnel blinded to case status. Genotyping, data entry and statistical analyses results were reviewed by two authors independently. Five percent samples were randomly selected to duplicate and yielded a 100% concordance.

### Statistics

The SAS software (version 9.2—SAS Institute, Cary, NC, USA) was used to test the association between DDH patients and control subjects. First of all, two-sided chi-squared tests were performed to determine the significance of differences in allelic frequencies and P<0.05 was considered statistically significant for Set B. The data of set A and set B was then combined using an additive, 2-tailed Cochran-Mantel-Haenszel model. Hardy-Weinberg equilibrium was calculated by chi-squared test in both control and case groups.

## Results

**Fig 1:**
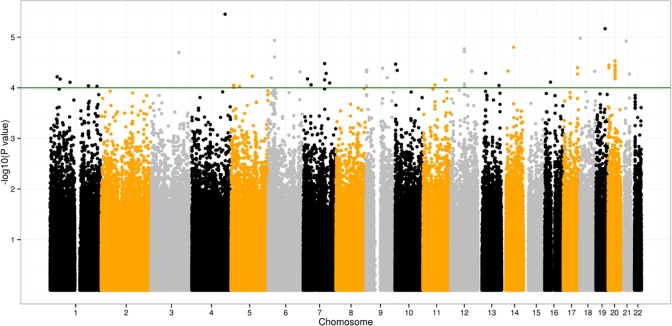
Genome-wide association results for DDH in Han Chinese populations. Scatter plot of *P* values on a —log_10_ scale from the logistic regression model in an additive model with adjustment for the top eigenvector. The green horizontal line represents *P* = 1.0 × 10^–4^.

In the DDH GWAS discovering stage, associations were assessed in an additive model using logistic regression analyses with adjustment for the top eigenvector (**Fig 1**). 51 SNPs showed significance of less than 10^–4^, and another 577 SNPs showed significance of less than 10^–3^ (S1 Table). From these 628 SNPs we found 21 SNPs at a same region, 20q11.22. *UQCC* and GDF5 genes located in this region. *GDF5* was previously reported as DDH susceptibility gene by our group. [7] In *UQCC*, all the 12 genotyped SNPs showed promising difference of allele frequencies. Details of the genotypes and allele distributions of cases and controls of the 12 SNPs were listed in Table 1. All of them showed significance of less than 5*10^–6^, with similar odds ratios ranging from 1.73 to 1.77 for the risk alleles (Table 1) rs6060373 was reported to be associated with body measurement traits and osteoarthritis, [18] and linkage Disequilibrium tests demonstrated rs6060373 had D’ value>0.9 to all the other 11 SNPs within *UQCC*. (S2 Fig) So we chose rs6060373 as the most promising locus and conducted a case-control analysis in an independent set of subjects (set B).

Distributions of genotypes of rs6060373 in both case and control groups were conformed to Hardy-Weinberg equilibrium in both set A and set B (p>0.1). Genotyping of rs6060373 in set A showed the minor allele A as a promising risk allele (p = 4.82*10^–7^). In set A, the odds ratio of allele A was 1.77 (Table 1). Genotyping of rs6060373 in another independent population consisting of 755 cases and 944 controls (Set B) produced another significant result (p = 0.0338) with an odds ratio of 1.18 for risk allele A (Table 2). Combining set A and set B, we identified a total p value of 3.63*10^–6^ with the odds ratio of 1.35 (1.19–1.53) for allele A.

**Table 2:** Association of rs6060373 in *UQCC* gene with DDH.

Population	Case					Control					P value for allele frequency	Odds ratio (95% CI)
	Genotype				Allele G frequency	Genotype				Allele G frequency		
	AA	AG	GG	Sum		AA	AG	GG	Sum			
**Set A**	260	109	17	386	0.19	285	226	47	558	0.29	4.82*10 ^**–7**^	1.77(1.41–2.21)
**Set B**	426	293	36	755	0.24	500	371	73	944	0.27	**0.0338**	1.18(1.01–1.38)
**Set A + Set B** ^**#**^	686	402	53	1141	0.22	785	597	120	1502	0.28	3.63*10 ^**–6**^	1.35(1.19–1.53)

Odds ratio shown is for allele A versus allele G.

^#^Set A and Set B were combined using an additive, 2-tailed Cochran-Mantel-Haenszel model.

## Discussion

We conducted the first GWAS for DDH and identified a new DDH susceptibility gene *UQCC*. Important roles of *UQCC* have been revealed in body measurement traits (e.g. height, skeletal frame and spine size) and osteoarthritis. [18] rs6060373 was highlighted in previous reports, and it had D’ value>0.9 to other 11 SNPs in *UQCC*. [21] So we chose rs6060373 for replication study on behalf of other 11 SNPs in *UQCC*, and it appeared to be a risk locus for DDH.

*UQCC* encodes a zinc-binding protein as a chaperone in mitochondrial respiratory chain to assembly Ubiquinol-cytochrome c reductase, of which activity is reduced in yeast lacking *UQCC*. [22] Interestingly, activity of ubiquinol-cytochrome c reductase is significantly reduced in cultured osteoarthritic chondrocytes compared to normal chondrocytes.[23] *UQCC* is identified as a target gene of FGF2 using induction gene trap approach in embryonic stem cells. FGF2 plays a vital role in chondrogenesis. [24] Overexpression of FGF2 results in dyschondroplasia and hence dwarfism in mice while FGF2 knock-out mice will have decreased bone density and develop accelerated osteoarthritis. [25–28] UQCC, repressed by FGF2, is likely to be involved in regulation of skeletal development and chondrogenesis by FGF2. However, further studies are needed to reveal its specific functional role in growth.

In conclusion, our study demonstrates common variants of *UQCC*, specifically rs6060373, are associated with DDH in Han Chinese population.

## Supporting Information

S1 Fig Quality-control was conducted at both the sample and single nucleotide polymorphism (SNP) levels as illustrated. (JPG)

S2 Fig Linkage Disequilibrium tests demonstrated all 12 SNPs within *UQCC* gene had a D’ value>0.9 to rs6060373. (JPG)

S1 Table Summary of loci associated with DDH in GWAS discovering stages. (DOCX)
