# Supplementary figures and images for: A Common Variant Of Ubiquinol-Cytochrome c Reductase Complex Is Associated with DDH
Source: PLoS One. 2015 Apr 7;10(4):e0120212. doi: 10.1371/journal.pone.0120212 (PMC4388640; doi:10.1371/journal.pone.0120212)

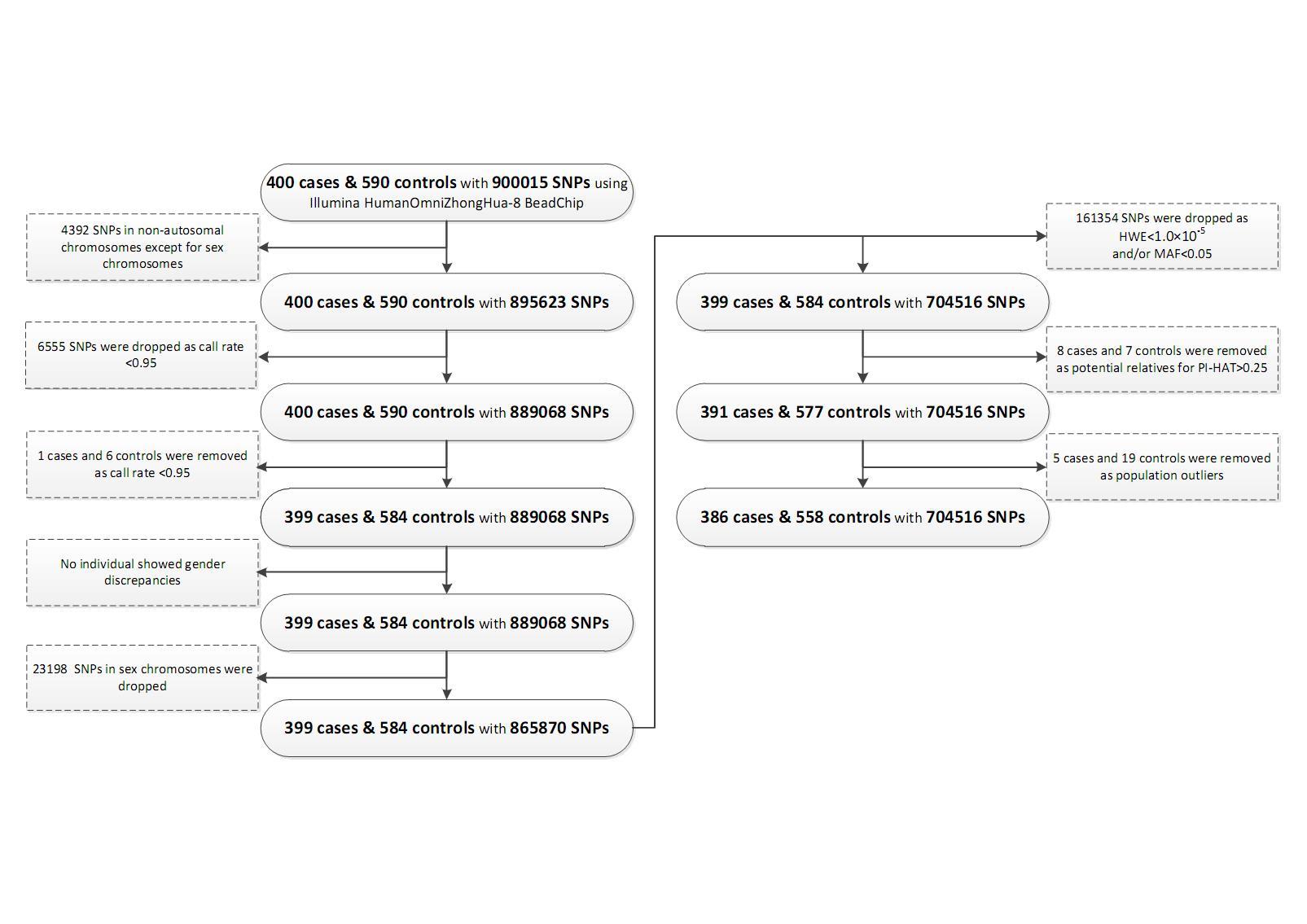

Supplement: S1 Fig — (JPG) [file pone.0120212.s001.jpg]

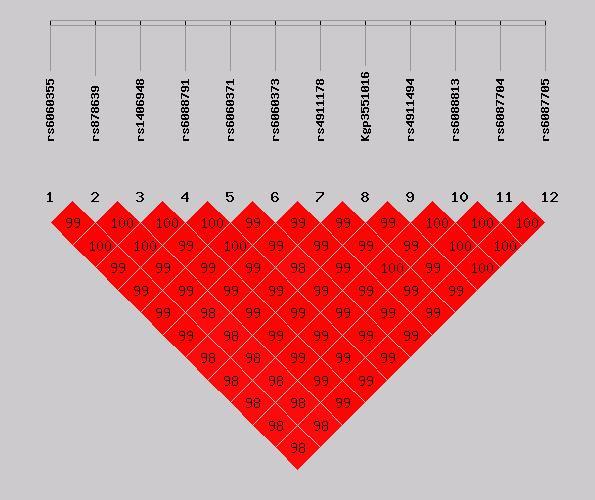

Supplement: S2 Fig — (JPG) [file pone.0120212.s002.jpg]
