# Supplementary material for: A Common Variant Of Ubiquinol-Cytochrome c Reductase Complex Is Associated with DDH
Source: PLoS One. 2015 Apr 7;10(4):e0120212. doi: 10.1371/journal.pone.0120212 (PMC4388640; doi:10.1371/journal.pone.0120212)
Supplement: S1 Table — (DOCX) [file pone.0120212.s003.docx]

|  |  |  | **Genotype distribution** | | **MAF** | |  | | |  |
| --- | --- | --- | --- | --- | --- | --- | --- | --- | --- | --- |
| **Chr.** | **Chr. Position** | **GENE** | **Cases** | **Controls** | **Cases** | **Controls** | **OR_add_^b^  95% CI** | | | **P_add_ Value^b^** |
|  |  |  |  |  |  |  |  | **Lower limit** | **Upper limit** |  |
| 1 | 38680372 | LINC01343 | 5/83/298 | 2/70/485 | 0.120 | 0.066 | 1.92 | 1.40 | 2.65 | 4.94E-05 |
| 1 | 49554476 | ABGL4 | 10/155/202 | 53/253/250 | 0.238 | 0.323 | 0.66 | 0.53 | 0.81 | 9.16E-05 |
| 1 | 191406367 |  | 24/202/116 | 117/299/142 | 0.365 | 0.478 | 0.63 | 0.52 | 0.77 | 3.30E-06 |
| 2 | 559913 |  | 124/187/41 | 145/290/121 | 0.382 | 0.478 | 1.48 | 1.22 | 1.80 | 5.72E-05 |
| 2 | 40142813 | SLC8A1-AS1 | 9/70/307 | 2/62/494 | 0.114 | 0.059 | 2.05 | 1.47 | 2.86 | 1.90E-05 |
| 3 | 39265616 | LOC102724104 | 6/87/293 | 3/77/478 | 0.128 | 0.074 | 1.83 | 1.35 | 2.49 | 9.79E-05 |
| 3 | 115327451 |  | 10/124/250 | 10/115/433 | 0.188 | 0.121 | 1.68 | 1.30 | 2.16 | 6.56E-05 |
| 3 | 158247867 | RSRC1 | 282/95/9 | 470/85/3 | 0.146 | 0.082 | 0.52 | 0.39 | 0.69 | 8.33E-06 |
| 4 | 162065899 |  | 25/179/150 | 105/282170 | 0.323 | 0.442 | 0.60 | 0.50 | 0.74 | 5.09E-07 |
| 5 | 104257922 |  | 1/20/330 | 2/82/469 | 0.031 | 0.078 | 0.38 | 0.24 | 0.62 | 5.01E-05 |
| 5 | 179616398 | HNRNPH1 | 105/180/101 | 93/270/194 | 0.495 | 0.591 | 1.47 | 1.22 | 1.77 | 3.93E-05 |
| 6 | 14988767 |  | 150/184/51 | 280/245/32 | 0.371 | 0.277 | 0.65 | 0.53 | 0.79 | 1.61E-05 |
| 6 | 26272829 | HIST1H2BI | 21/130/235 | 15/130/413 | 0.223 | 0.143 | 1.71 | 1.35 | 2.17 | 8.49E-06 |
| 6 | 26289310 |  | 22/129/235 | 15/130/413 | 0.224 | 0.143 | 1.73 | 1.36 | 2.19 | 6.20E-06 |
| 6 | 26293841 |  | 239/125/22 | 417/127/14 | 0.219 | 0.139 | 0.58 | 0.45 | 0.73 | 5.93E-06 |
| 6 | 26328365 | LOC101928743 | 16/120/250 | 12/119/427 | 0.197 | 0.128 | 1.67 | 1.30 | 2.14 | 5.31E-05 |
| 6 | 26330492 | LOC101928743 | 21/129/236 | 15/133/410 | 0.222 | 0.146 | 1.66 | 1.31 | 2.11 | 2.45E-05 |
| 6 | 26331969 | LOC101928743 | 21/129/236 | 15/133/410 | 0.222 | 0.146 | 1.66 | 1.31 | 2.11 | 2.45E-05 |
| 6 | 26338056 | BTN3A2 | 21/129/236 | 15/133/410 | 0.222 | 0.146 | 1.66 | 1.31 | 2.11 | 2.45E-05 |
| 6 | 30788191 | LINC00243 | 3/57/326 | 0/32/525 | 0.082 | 0.029 | 3.00 | 1.94 | 4.65 | 2.52E-07 |
| 6 | 31161571 | HCG27 | 172/181/7 | 200/274/67 | 0.271 | 0.377 | 1.63 | 1.33 | 2.00 | 2.92E-06 |
| 6 | 31335403 | HLA-B | 167/166/16 | 188/286/80 | 0.284 | 0.403 | 1.70 | 1.39 | 2.09 | 2.93E-07 |
| 6 | 31374579 | MICA | 230/143/4 | 283/232/39 | 0.2 | 0.28 | 1.55 | 1.24 | 1.94 | 9.65E-05 |
| 6 | 31461509 | MICB | 63/205/90 | 178/268/109 | 0.462 | 0.562 | 0.67 | 0.55 | 0.81 | 3.08E-05 |
| 6 | 101731443 |  | 90/207/59 | 94/283/180 | 0.456 | 0.577 | 1.63 | 1.35 | 1.96 | 4.77E-07 |
| 7 | 20572445 | ABCB5 | 13/129/243 | 42/233/282 | 0.201 | 0.285 | 0.63 | 0.51 | 0.79 | 4.20E-05 |
| 7 | 111439686 | IMMP2L | 133/196/19 | 167/277/112 | 0.336 | 0.451 | 1.62 | 1.33 | 1.97 | 1.51E-06 |
| 10 | 11048244 |  | 20/146/220 | 59/264/235 | 0.241 | 0.342 | 0.61 | 0.50 | 0.75 | 2.41E-06 |
| 11 | 5026767 |  | 37/174/152 | 116/257/184 | 0.342 | 0.439 | 0.66 | 0.55 | 0.80 | 3.16E-05 |
| 11 | 49671608 | LOC440040 | 339/20/0 | 469/74/5 | 0.028 | 0.077 | 2.90 | 1.76 | 4.76 | 1.26E-05 |
| 12 | 67357764 |  | 5/86/295 | 0/76/482 | 0.124 | 0.068 | 1.94 | 1.42 | 2.67 | 3.02E-05 |
| 12 | 67365203 |  | 279/85/4 | 485/72/0 | 0.12 | 0.065 | 0.50 | 0.37 | 0.70 | 2.49E-05 |
| 13 | 37168075 |  | 4/67/315 | 15/151/392 | 0.097 | 0.162 | 0.56 | 0.42 | 0.74 | 5.02E-05 |
| 14 | 84097837 |  | 98/200/72 | 102/270/184 | 0.465 | 0.574 | 1.55 | 1.29 | 1.87 | 4.36E-06 |
| 15 | 58446034 | AQP9 | 0/23/332 | 5/95/452 | 0.032 | 0.095 | 0.32 | 0.20 | 0.51 | 3.70E-07 |
| 18 | 2451449 |  | 32/175/146 | 30/198/327 | 0.339 | 0.232 | 1.69 | 1.37 | 2.08 | 7.93E-07 |
| 19 | 16001740 | CYP4F2 | 29/183/149 | 106/274/175 | 0.334 | 0.438 | 0.64 | 0.53 | 0.78 | 8.86E-06 |
| 19 | 44875317 | GALR1 | 69/216/77 | 202/253/100 | 0.489 | 0.592 | 0.66 | 0.55 | 0.80 | 1.50E-05 |
| 20 | 33890061 | UQCC | 17/110/259 | 46/226/286 | 0.187 | 0.285 | 0.58 | 0.46 | 0.72 | 1.04E-06 |
| 20 | 33894463 | UQCC | 259/110/17 | 284/228/46 | 0.187 | 0.287 | 1.75 | 1.40 | 2.19 | 6.85E-07 |
| 20 | 33905619 | UQCC | 17/109/260 | 46/226/286 | 0.185 | 0.285 | 0.57 | 0.46 | 0.71 | 7.35E-07 |
| 20 | 33907909 | UQCC | 260/109/17 | 285/226/46 | 0.185 | 0.285 | 1.76 | 1.41 | 2.20 | 6.57E-07 |
| 20 | 33913322 | UQCC | 18/107/260 | 47/225/286 | 0.186 | 0.286 | 0.57 | 0.46 | 0.71 | 6.93E-07 |
| 20 | 33914208 | UQCC | 260/109/17 | 285/226/47 | 0.185 | 0.287 | 1.77 | 1.41 | 2.21 | 4.82E-07 |
| 20 | 33952620 | UQCC | 258/111/17 | 285/227/46 | 0.188 | 0.286 | 1.73 | 1.39 | 2.16 | 1.19E-06 |
| 20 | 33954913 | UQCC | 258/110/17 | 286/223/48 | 0.187 | 0.286 | 1.74 | 1.40 | 2.18 | 8.77E-07 |
| 20 | 33971914 | UQCC | 259/110/17 | 286/226/47 | 0.187 | 0.285 | 1.74 | 1.39 | 2.17 | 1.04E-06 |
| 20 | 33975181 | UQCC | 259/110/17 | 284/226/46 | 0.187 | 0.286 | 1.75 | 1.40 | 2.18 | 8.32E-07 |
| 20 | 34001058 | UQCC | 259/110/17 | 286/226/46 | 0.187 | 0.285 | 1.74 | 1.39 | 2.17 | 1.04E-06 |
| 20 | 34001250 | UQCC | 259/110/17 | 286/226/46 | 0.187 | 0.285 | 1.74 | 1.39 | 2.17 | 1.04E-06 |
| 21 | 24929472 |  | 52/184/115 | 153/273/131 | 0.41 | 0.52 | 0.64 | 0.53 | 0.78 | 5.52E-06 |

**Supplementary Table 1** Summary of loci associated with DDH in GWAS discovering stages

| ^a^Individuals homozygous for the minor allele/heterozygous/homozygous for the major allele. ^b^OR_add_ (95% confidence interval(CI)) and *P*_add_ values were derived from logistic regression analysis in the additive model with adjustment for the top eigenvector. |
| --- |
